# Comparative quantitative trait loci for silique length and seed weight in *Brassica napus*

**DOI:** 10.1038/srep14407

**Published:** 2015-09-23

**Authors:** Ying Fu, Dayong Wei, Hongli Dong, Yajun He, Yixin Cui, Jiaqin Mei, Huafang Wan, Jiana Li, Rod Snowdon, Wolfgang Friedt, Xiaorong Li, Wei Qian

**Affiliations:** 1 College of Agronomy and Biotechnology, Southwest University, Chongqing 400716, China; 2 Department of Plant Breeding, IFZ Research Centre for Biosystems, Land Use and Nutrition, Justus Liebig University, Heinrich-Buff-Ring 26-32, 35392 Giessen, Germany

## Abstract

Silique length (SL) and seed weight (SW) are important yield-associated traits in rapeseed (*Brassica napus*). Although many quantitative trait loci (QTL) for SL and SW have been identified in *B. napus*, comparative analysis for those QTL is seldom performed. In the present study, 20 and 21 QTL for SL and SW were identified in doubled haploid (DH) and DH-derived reconstructed F_2_ populations in rapeseed, explaining 55.1–74.3% and 24.4–62.9% of the phenotypic variation across three years, respectively. Of which, 17 QTL with partially or completely overlapped confidence interval on chromosome A09, were homologous with two overlapped QTL on chromosome C08 by aligning QTL confidence intervals with the reference genomes of Brassica crops. By high density selective genotyping of DH lines with extreme phenotypes, using a *Brassica* single-nucleotide polymorphism (SNP) array, the QTL on chromosome A09 was narrowed, and aligned into 1.14-Mb region from 30.84 to 31.98 Mb on chromosome R09 of *B. rapa* and 1.05-Mb region from 27.21 to 28.26 Mb on chromosome A09 of *B. napus*. The alignment of QTL with Brassica reference genomes revealed homologous QTL on A09 and C08 for SL. The narrowed QTL region provides clues for gene cloning and breeding cultivars by marker-assisted selection.

Rapeseed (*Brassica napus*), which originated from spontaneous hybridization between *B. rapa* (AA) and *B. oleracea* (CC)^1^, is one of the most important oilseed crops worldwide. The silique is not only a photosynthetic source organ, supplying approximately 30% of the dry matter of the silique and seed^2^,^3^,^4^, but also an important sink organ that imports carbohydrates^5^. Moreover, the silique also serves to coordinate seed filling, regulates the reallocation of reserves, and protects seeds against biotic and abiotic stresses^6^. The silique length (SL) is significantly positively correlated with variation in seed weight (SW)^7^,^8^,^9^, one of the three components of plant grain yield (number of siliques per plant, number of seeds per silique, and seed weight). Therefore, long silique is a desirable trait in rapeseed breeding programs^7^,^10^.

Many research efforts have been focused on dissecting quantitative trait loci (QTL) for SL and SW. M M ore than 20 QTL for SL distributed on 15 chromosomes and more than 80 QTL for SW distributed on 19 chromosomes have been identified in *B. napus*^11^,^12^,^13^,^14^,^15^,^16^,^17^,^18^,^19^. However, comparison among the QTL is difficult because different populations and molecular marker systems were adopted in these studies. With the release of reference genomes for *Brassica* crops, such as *B. napus*, *B. rapa* and *B. oleracea*^20^,^21^,^22^, it is feasible to conduct comparative genome analysis in *Brassica* crops.

Traditional QTL mapping has a number of disadvantages that include longer research time and lower mapping resolutions. With the rapid development of next-generation sequencing (NGS) technologies, some new strategies were proposed to take advantages of the power of high-throughput genotyping, e.g. QTL-seq approach^23^, where two DNA bulks of progenies with extreme phenotypic values (‘Highest’ and ‘Lowest’ bulks) are whole-genome re-sequenced to produce amount of reads, and the QTL are identified via screening the genomic regions that show high differences of reads between ‘Highest’ and ‘Lowest’ bulks. In comparison with the traditional QTL mapping, the NGS-aided strategy provides a simple and effective alternative to rapidly identify QTL of interest by genotyping small number of samples from two sets of individuals with distinct or opposite extreme phenotypes^23^,^24^. By using the NGS-aided strategy, a few QTL of the interested traits have been successfully identified in yeast^23^,^25^,^26^,^27^, rice^24^,^28^,^29^, *Arabidopsis thaliana*^30^, sunflower^31^, cucumber^32^, wheat^33^, tomato^34^ and chickpea^35^.

In this study, the strategies of conventional QTL mapping and high-throughput genotyping were combined to dissect QTL of SL and SW in a doubled haploid (DH) population and its reconstructed F_2_ (RC-F_2_) population of rapeseed. Based on the alignment of SSR markers to the reference genomes of *Brassica* crops, the genetic region on chromosome A09 where the 17 overlapped QTL of SL and SW on chromosome A09 enriched, was revealed to be homologous with the overlapped QTL for SL on chromosome C08. The major QTL region on chromosome A09 was aligned to a ~1 Mb region on the reference genome of *B. rapa* and *B. napus* with high density SNP array.

## Results

### Variation in silique length and seed weight

**Figure 1:**
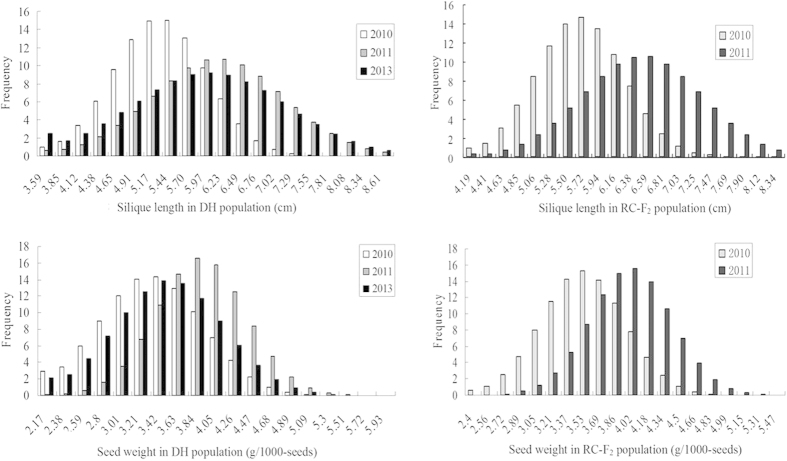
Frequency distributions of silique length and seed weight in the DH population in 2010, 2011 and 2013, and the RC-F_2_ population in 2010 and 2011.

The semi-winter parental line ‘SWU07’ exhibited higher SL and SW values than the winter parental line ‘Express’. Wide variation was detected in both the DH and RC-F_2_ populations for SL and SW across the years analyzed (Fig. 1). The field performance of the 233 RC-F_2_ lines, with an average SL of 6.13 ± 0.71 cm and an average SW of 3.77 ± 0.38 g, was superior to that of 261 DH lines, which had an average SL of 5.67 ± 0.81 cm and an average SW of 3.47 ± 0.46 g. The normal distribution for SL and SW in both populations suggested that SL and SW were controlled by multiple genes (Fig. 1).

The ANOVA results showed significant differences among genotypes, years and genotype-by-year interactions for SL and SW in the two populations (P < 0.01) (Table S1). High broad-sense heritability was detected for SL and SW (average of 83.79% for SL and 79.13% for SW). The significant and positive correlation between SL and SW in both populations (r = 0.49 and 0.34 in the DH and RC-F_2_ populations, respectively; P < 0.01) suggested that long silique had the potential to increase SW.

### QTL analysis

**Figure 2:**
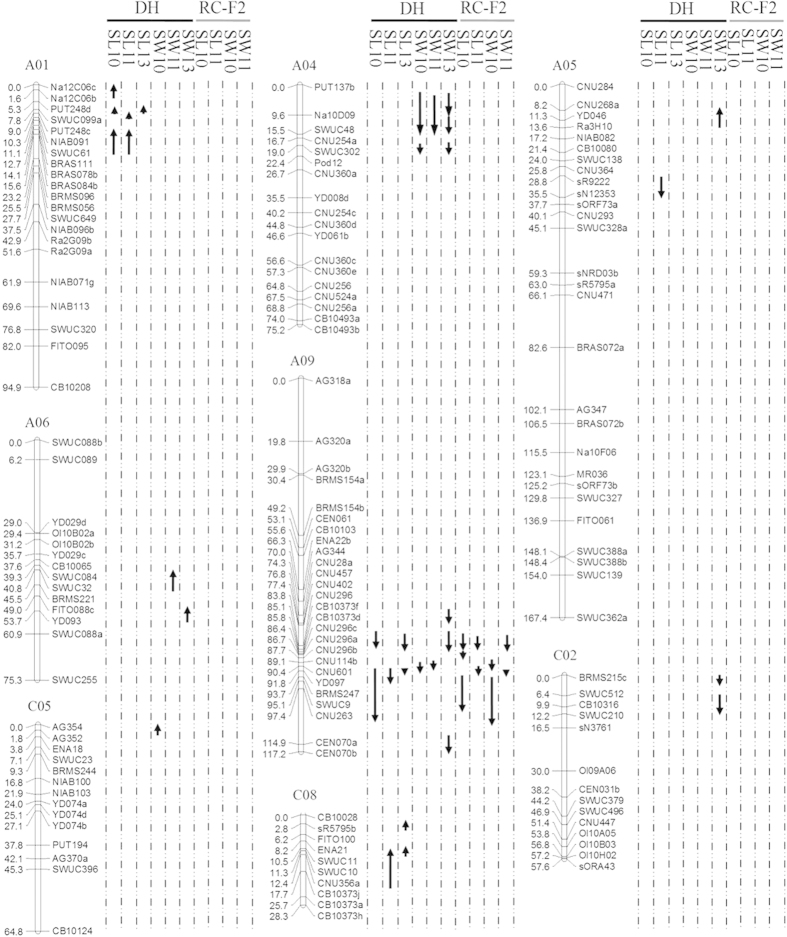
QTL for silique length and seed weight in the DH population in 2010, 2011 and 2013, and the RC-F_2_ population in 2010 and 2011. Upward and downward arrows indicate additive effects of QTL in alleles contributed by the parents ‘Express’ and ‘SWU07’, respectively.

20 QTL were located on chromosomes A01, A05, A09, and C08 for SL in the DH and RC-F_2_ populations across years, totally explaining 55.1–75.7% of the phenotypic variation, whereas 21 QTL were detected for SW on A04, A05, A06, A09, C02, and C05, totally explaining 24.4–62.9% of the phenotypic variation in the DH and RC-F_2_ populations across years (Table 1, Fig. 2). Among these QTL, 17 QTL for SL and SW were enriched in a region on chromosome A09 from 80 cM to 107.5 cM with partially or completely overlapped confidence interval. The direction of positive additive effect for these QTL was consistent from parental line ‘SWU07’ (Fig. 2). No digenic interaction with significant and high effect was found for SL and SW, although nine and ten additive by additive interactions were detected for SL and SW with minor effects, total explaining 2.78% and 4.14% of the phenotypic variation in DH and RC-F_2_ populations, respectively. Together the positive correlation between SL and SW and overlapped confidence intervals of QTL, indicated that some common genetic factors might regulate both SL and SW.

**Figure 3:**
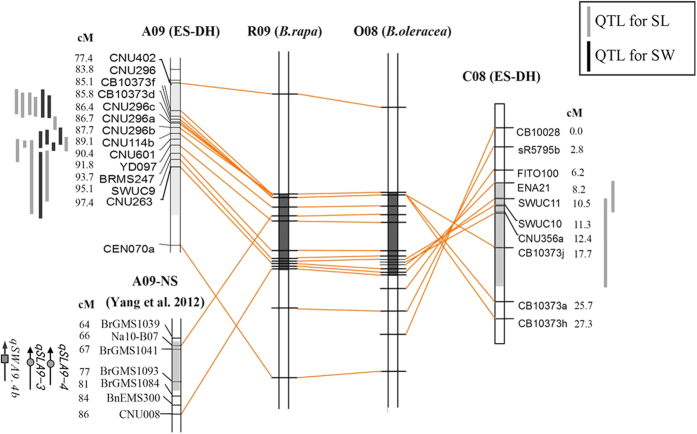
Comparative analysis of QTL for silique length and seed weight on chromosomes A09 and C08 via alignment of the SSR loci linked with the QTL to chromosomes R09 and O08 of the reference genomes of *B. rapa* and *B. oleracea*, respectively. ES-DH was derived from the cross between ‘Express’ and ‘SWU07’ in this study, and the NS population was from the study of Yang *et al*. (2012). Lines beside linkage groups represent QTL for silique length and seed weight, respectively. The light-grey highlighted regions on the linkage groups showed the QTL intervals, and the dark-grey highlighted regions on the genomes exhibited the co-location physical regions of the QTL on A09 and C08.

Given the high degree of synteny between A09 and C08 (http://genomevolution.org/wiki/index.php/Brassica_oleracea_v._Brassica_rapa), the microsynteny between QTL of SL on A09 and C08 was compared by aligning QTL intervals with the reference genomes of *B. rapa* and *B. oleracea* (Fig. 3). The interval of 17 overlapped QTL for SL and SW on chromosome A09, linked with 9 molecular markers (CNU402 ∼ CNU263), was aligned to the regions from 26.7 Mb to 33.7 Mb on chromosome R09 of the reference genome of *B. rapa* and from 27.2 Mb to 35.6 Mb on chromosome O08 of *B. oleracea*, whereas two overlapped QTL for SL on chromosome C08, linked with 5 molecular markers (ENA201 ∼ CB10373), were corresponded to the physical positions from 30.8 Mb to 33.8 Mb on chromosome R09 of *B. rapa* and from 31.7 Mb to 35.7 Mb on chromosome O08 of *B. oleracea* (Fig. 3; Table S2). The co-localization of QTL suggests homoeologous duplicated QTL for SL on chromosome A09 and C08 in rapeseed.

In order to narrow the QTL region in A09, 25 DH lines with extreme performance of SL and SW, were screened by 293 SSR markers to evaluate genetic background. Five and six lines were finally chosen to construct the ‘Large’ and ‘Short’ groups, respectively. Thus the two groups shared the same genetic background except for the region of QTL on chromosome A09. The average values for SL and SW in the ‘Large’ group (SL 7.46 cm and SW 4.18 g) were significantly higher (*p* < 0.01) than the ‘Small’ group (SL 3.97 and SW 3.04 g). Each individual from two groups was subjected to high-density selective genotyping using the *Brassica* 60 K SNP Bead Chip Array. Of the 52,157 SNP markers in the *Brassica* SNP array, 41,645 (80%) could be detected among 11 DH lines, while only 2751 (6.61%) SNPs were polymorphic between the ‘Large’ group and ‘Small’ group, indicating their similar genetic background.

**Figure 4:**
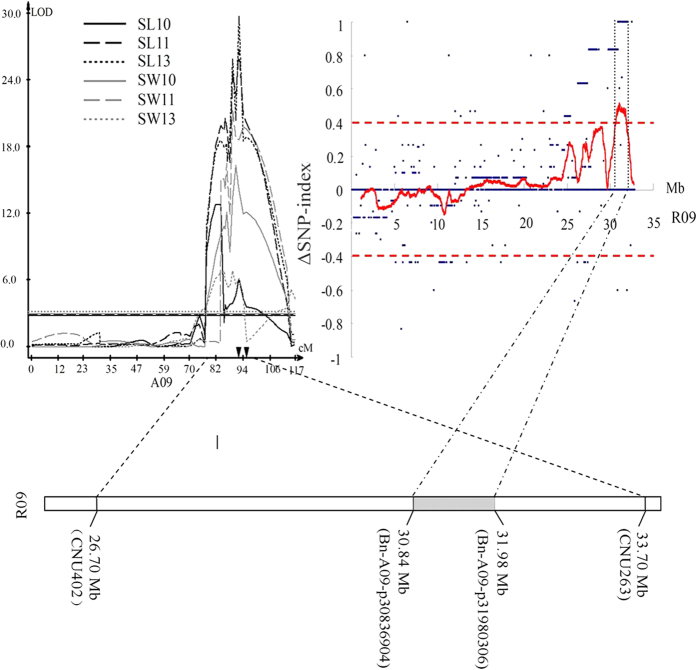
Mapping of QTL on A09 using the approach of SNP array analysis. The QTL on A09 detected in the DH population was shown in the left graph, the small triangles on the *x*-axis represented the location of the region-specific SSR markers. The ΔSNP-index values of chromosome R09 were plotted in the right graph, and the dotted line indicated the threshold level at *p* = 0.01. The diagram below these two graphs showed the location of the reference genome region on R09 of *Brassica rapa* corresponding with the QTL.

The Δ (SNP-index) of each SNP locus was calculated by subtraction of SNP-index of the ‘Large’ group from the ‘Small’ group, and the Δ (SNP-index) trends were visualized by means of a sliding window (Fig. 4). A single peak region with a significant (*p* < 0.01) and high value of Δ (SNP-index), harboring 45 SNP markers (Table S3), was identified (Fig. 4). It corresponded to 1.14-Mb region from 30.84 to 31.98 Mb on chromosome R09 of *B. rapa* (Fig. 4), and 1.05-Mb region from 27.21 to 28.26 Mb on chromosome A09 of *B. napus*, which were located into the interval of QTL on A09 detected by conventional QTL mapping. A total of 241 and 225 genes were harbored in these two regions, respectively. Of which, 69.71% (168/241) genes on the chromosome R09 of *B. rapa* were orthologous to 73.78% (166/225) genes on the chromosome A09 of *B. napus*, suggesting the homology between two regions.

To confirm the narrowed region harboring QTL, ten region-specific SSR (RS-SSR) markers were developed according to the genomic sequence of the regions in reference genome of *B. rapa* and *B. napus*. Of which, two RS-SSR markers (CY-04 and CY-10) that exhibited polymorphisms between two parents were successfully mapped to the confidence interval of the QTL on A09 in DH population (Fig. 4), further indicating the consistency between QTL mapping and NGS-aided studies.

## Discussion

QTL mapping is the main approach for genetic dissection of quantitative traits, which provides the start point for map-based cloning of related genes and marker-assisted selection (MAS) in plant breeding. Although QTL mapping for silique traits have been reported^11^,^12^,^13^,^14^,^15^,^16^,^17^,^18^,^19^, single population was almost adopted in these studies. In this study, two related populations, DH and its derived RC-F_2_ populations were used. The RC-F_2_ population holds unique characteristics with normal F_2_ population if genotypic selection did not exist in the process of microspore culture, but has advantages over normal F_2_ population. For example, it permits the possibility of replicated experiments in multiple years or environments. The major QTL on A09 were repeatedly detected in both populations, indicating the credibility of the QTL on A09.

More than 100 QTL have been detected for SL and SW in rapeseed^11^,^12^,^13^,^14^,^15^,^16^,^17^,^18^,^19^, but the detailed comparative or functional analyses of these QTL have not been reported. In this study, we assigned QTL regions onto the reference genomes of *Brassica* crops through BLAST analysis of markers linked to the QTL. This enabled us to detect homeologous duplicated QTL for SW and SL on *B. napus* chromosomes A09 and C08. In order to test the power of this approach, we compared our result with the study of Yang *et al*. (2012), who also identified a major QTL region for SL and SW on chromosome A09 in *B. napus* using different markers with this study^16^. By aligning markers in the flank of the QTL (Na10-B07 and CNU008) to the reference genome of *B. rapa*, the confidence intervals of the QTL were aligned to the genomic region on chromosome R09 of *B. rapa* from 30.1 to 32.2 Mb, which partially overlapped with that of QTL detected in the current study (Fig. 3). These findings supported the credibility of QTL on A09 controlling SL and SW.

The conventional approach to narrow QTL regions is a laborious process that requires the development of DNA markers and the generation of a large number of advanced-generation progenies. These requirements limit its use because they are time consuming and costly, particularly in annual crop species like rapeseed. With the release of reference genomes for some species and advances in NGS technology, several novel strategies for QTL mapping have been proposed with the use of high-throughput genotyping, such as microarray-based genotyping or massively parallel sequencing. In the present study, the process of QTL-seq was followed with slight modification, i.e. a high-density Brassica SNP array, instead of resequencing of bulks in QTL-seq, was used to genotype DH lines with the extreme trait values, and the QTL were successfully anchored to a ~1 Mb region on chromosome R09 of *B. rapa* and A09 of *B. napus*. Given the high-throughput nature and low cost of the SNP array, this modified approach will dramatically accelerate the process of QTL fine-mapping in a cost-effective manner.

## Conclusions

We identified 20 and 21 QTL for SL and SW in DH and RC-F_2_ populations of *B. napus* across three years. A significant and positive correlation between SL and SW, and overlapped confidence intervals among partial QTL for SL and SW detected in this study suggested long silique has the potential to increase SW. The major QTL for SL on chromosome A09 and C08 were aligned to the same region of the reference genomes of Brassica crops, suggesting they are homologous QTL. By high density selective genotyping of DH lines with extreme phenotypes, using a *Brassica* single-nucleotide polymorphism (SNP) array, the region of major QTL on chromosome A09 was aligned to a ~1 Mb region on the reference genome of *B. rapa* and *B. napus*.

## Methods

### Plant materials and phenotypic evaluation

A *B. napus* DH population, consisting of 261 lines, was developed from a cross between the European winter cultivar ‘Express’ (female) and Chinese semi-winter line ‘SWU07’ (male). Two parental lines showed diversity in SL and SW. Two rounds of random crosses between DH lines were performed to reconstruct 233 RC-F_2_ lines. Each DH line was used once each round.

The two populations, together with the parental lines, were grown in the experimental field of Southwest University, Chongqing, China, in 2010, 2011 and 2013. A randomized complete block design was employed with two replications. Each plot with the acreage of 2.5 m^2^ consisted of 30 plants, with 30 cm spacing between rows and 20 cm spacing within rows.

Ten well-developed siliques per plant at the base of the inflorescence were collected, and siliques from ten individuals in the center of each plot were pooled to measure silique length and weight at maturity.

### Statistical analysis

Analysis of variance (ANOVA) was performed using the GLM procedure of SAS (SAS Institute, 2000). The broad-sense heritability (H^2^) was calculated as: 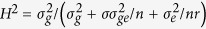, where 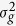, 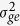 and 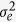 are estimates of the variances of genotype, genotype × environment interactions, and error, respectively, *n* is the number of environments, and *r* is the number of replications per environment^36^. Pearson’s correlation coefficients between traits of interest were calculated using the CORR procedure of SAS^37^.

### QTL analysis

DNA isolation, development of molecular markers and construction of genetic linkage groups were described in previous study^38^, where 293 SSR markers were arranged into 19 *B. napus* chromosomes, spanning a genetic distance of 1,188 cM with an average distance of 4.05 cM between adjacent markers. Besides, the sequence of target region was downloaded from the reference genomes (http://brassicadb.org/brad/index.ph), and screened for SSR loci using the software “SSR Locator”^39^. Ten region-specific SSR markers were developed according to the genome sequence of the target QTL region. The genotypes for RC-F_2_ lines were deduced from the band patterns of their parental lines. A genotype score of ‘1’ was given to a RC-F_2_ line if the SSR marker was present in at least one of the parental lines, while the RC-F_2_ line was assigned a score of ‘0’ if the marker was absent in both parents. QTL detection was performed with the composite interval mapping (CIM) procedure of the software WinQTL Cartographer 2.5^40^. A 1,000-permutation test was performed to estimate a significance threshold of the test statistic for a QTL based upon a 5% experiment-wise error rate^41^. The genome-wide digenic interactions were estimated using QTL mapper V2.0 software^42^.

The SL and SW QTL regions were aligned to the reference genomes of *B. rapa* (version 1.5) (http://brassicadb.org/brad/index.ph), *B. napus* (http://www.genoscope.cns.fr/brassicanapus/cgi-bin/gbrowse/colza/) and *B*. *oleracea* (version 1.0) (http://www.ocri-genomics.org/bolbase/) by BLAST analysis of the sequences of SSR markers or SSR primers linked with QTL, with slight modification of the method of Cai *et al*. (2012)^43^. The alignment criteria were set to allow three mismatches and one gap for a given primer pair. When a marker had multiple amplification loci on the same chromosome, an accurate position for a particular locus was determined manually by referring to the physical positions of its upstream and downstream markers.

### SNP array analysis

In order to narrow the confidence intervals of major QTL in A09, two pools of DH lines with extreme performance were constructed for SNP array analysis. Briefly, The DH lines with extreme performance of SL and SW were first evaluated by 293 SSR markers for evaluating genetic background. And the samples which shared the same genetic background except for QTL on chromosome A09, were selected to construct the ‘Large’ group (extremely long siliques and high seed weight) and ‘Small’ group (extremely short siliques and low seed weight). Each DH lines of the two groups was genotyped with the *Brassica* 60 K SNP Bead Chip Array (Illumina Inc., CA, USA), together with the parental line ‘SWU07’. The single-nucleotide polymorphism (SNP) loci were aligned to the reference genomes of *B. rapa* (version 1.5) (http://brassicadb.org/brad/index.ph) and *B. napus* (http://www.genoscope.cns.fr/brassicanapus/cgi-bin/gbrowse/colza/)

The process of chip array analysis was performed in accordance with the method of Takagi *et al*. (2013) with slight modification^23^. The Δ (SNP-index) of each locus was calculated by subtraction of SNP-index of ‘large’ group from ‘small’ group with the formula, Δ (SNP-index) = *k*/5 − *j*/6, where *k* and *j* are the number of accessions that exhibit a consistant genotype from ‘SWU07’ in the ‘Large’ and ‘Small’ groups, respectively. A sliding window analysis was applied to generate Δ (SNP-index) plots with a window size of 80 SNP and increment of 1 SNP. 1,000 random resamplings were performed to estimate a significance threshold of the test statistic for a QTL based upon a 1% experiment-wise error. Thus the statistical confidence intervals under the null hypothesis of no QTL were determined at a significance level of *p* = 0.01

## Additional Information

**How to cite this article**: Fu, Y. *et al*. Comparative quantitative trait loci for silique length and seed weight in *Brassica napus*. *Sci. Rep*. **5**, 14407; doi: 10.1038/srep14407 (2015).

## Supplementary Material

Supplementary Information

## Figures and Tables

**Table 1:** QTL associated with silique length and seed weight in the DH and RC-F_2_ populations of *B. napus*.

Putative QTL	Linkage group^a^	DH population (values for 2010/2011/2013)				RC-F2 population (values in 2010/ in 2011)			
		Position^b^ (cM)	LOD^c^	A^d^	*R*^2^ (%)^e^	Position (cM)	LOD	A	*R*^2^ (%)
*qSL*1-1	A1	0–3.6/−/−	3.95/−/−	0.16/−/−	5.0/−/−	−/−	−/−	−/−	−/−
*qSL*1-2	A1	7.5–8.3/−/6.6−8.7	4.83/−/3.46	0.17/−/0.21	6.1/−/3.38	−/−	−/−	−/−	−/−
*qSL*1-3	A1	13.9–20.2/14.1–20.1/-	5.24/7.25/−	0.18/0.27/−	6.6/7.2/−	−/−	−/−	−/−	−/−
*qSL*1-4	A1	−/9.9–10.5/−	−/9.1/−	−/0.29/−	−/8.9/−	−/−	−/−	−/−	−/−
*qSL*5-1	A5	−/28.8–35.2/−	−/4.59/−	−/−0.22/−	−/4.9/−	−/−	−/−	−/−	−/−
*qSL9-1*	A9	80–83.8/−/81.5–85.8	12.85/−/18.59	−0.3/−/−0.59	19.5/−/25.68	81.9—85/82.3–84.5	6.19/13.27	−0.4/−0.61	18.3/24.8
*qSL*9-2	A9	−/−/−	−/−/−	−/−/−	−/−/−	85.8–88.5/−	10.85−	−0.5/−	27.8/−
*qSL*9-3	A9	90.4–104.8/91.5–95.7/91.6–92	11.18/26.77/29.72	−0.23/−0.55/−0.71	17.9/31.2/37.4	93.7−104.3/90.4–92.3	8.64/18.2	−0.47/−0.71	29.7/33.3
*qSL*18-1	C8	−/−/1.3–4.8	−/−/3.09	−/−/0.21	−/−/3.11	−/−/	−/−/	−/−/	−/−/
*qSL*18-2	C8	−/10.4–21.7/9.8–12.4	−/3.99/4.75	−/0.19/0.25	−/3.8/4.7	−/−	−/−	−/−	−/−
**Total**					**55.1/56/74.3**				**75.7/58.1**
*qSW*4-1	A04	2.6–15.5/3.3–14.8/2.8–9.6	3.4/3.23/4.87	−0.12/−0.09/−0.17	4.58/3.36/7.5	−/−	−/−	−/−	−/−
*qSW*4-2	A04	−/−/9.6–15.5	−/−/5.2	−/−/−0.15	−/−/6.54	−/−	−/−	−/−	−/−
*qSW*4-3	A04	18.6–19.7/−/17.9–21	3.19/−/2.87	−0.12/−/−0.13	3.99/−/4.49	−/−	−/−	−/−	−/−
*qSW*5-1	A05	−/−/7.6–13.6	−/−/2.9	−/−/0.11	−/−/3.3	−/−	−/−	−/−	−/−
*qSW*6-1	A06	−/−/40.7–46.5	−/−/2.92	−/−/0.09	−/−/3.32	−/−	−/−	−/−	−/−
*qSW*6-2	A06	−/−/52.5–56.5	−/−/3.65	−/−/0.14	−/−/4.39	−/−	−/−	−/−	−/−
*qSW*9-1	A09	−/−/72.9–76.8	−/−/3.55	−/−/−0.14	−/−/5.19	−/−	−/−	−/−	−/−
*qSW*9-2	A09	−/−/80.8–86.2	−/−/6.85	−/−/−0.19	−/−/8.65	−/82.6–84.9	−/−13	−/−0.3	−/23.73
*qSW*9-3	A09	89.1–91.9/88.9–90.2/−	16.34/21.31/−	−0.28/−0.27/−	22.93/27.36/−	87.7–91.4/91.5–92.2	4.73/16.36/−	−0.23/−0.32	10.31/27.83/−
*qSW*9-4	A09	−/−/−	−/−/−	−/−/−	−/−/−	93.5–107.5/−	4.13/−	−0.24/−	14.04/−
*qSW*9-5	A09	−/−/113.1–117	−/−/5.21	−/−/−0.16	−/−/6.36	−/−	−/−	−/−	−/−
*qSW*12-1	C02	−/−/0–2.3	−/−/5.05	−/−/−0.16	−/−/6.15	−/−	−/−	−/−	−/−
*qSW*12-2	C02	−/−/6.5–11.9	−/−/2.69	−/−/−0.11	−/−/3.35	−/−	−/−	−/−	−/−
*qSW*15-1	C05	0–3/−/−	−/−/3.02	−/−/0.11	−/−/3.69	−/−	−/−	−/−	−/−
**Total**					**31.5/30.7/62.91**				**24.4/51.6**

^a^A,C followed by a number designates the linkage group where the QTL was detected.

^b^Length of one LOD score confidence interval.

^c^Peak effect of the QTL (LOD, limit of detection).

^d^Additive effect: positive additivity indicates that the QTL allele originated from the parental line Express; negative additivity indicates that the QTL allele originated from the parental line SWU07.

^e^Percentage of the phenotypic variance explained by each QTL.
