# Supplementary material for: Comparative quantitative trait loci for silique length and seed weight in Brassica napus
Source: Sci Rep. 2015 Sep 23;5:14407. doi: 10.1038/srep14407 (PMC4585775; doi:10.1038/srep14407)
Supplement: Supplementary Information [file srep14407-s1.doc]

Comparative QTL for silique length and seed weight in ***Brassica napus***

Ying Fu^1,2^, Dayong Wei^1^, Hongli Dong^1^, Yajun He^1^, Yixin Cui^1^, Jiaqin Mei^1^, Huafang Wan^1^, Jiana Li^1^, Rod Snowdon^2^, Wolfgang Friedt^2^, Xiaorong Li^1^ and Wei Qian^1^*

Table S1 ANOVA for SL and SW in DH population in 2010, 2011 and 2013, and RC-F_2_ population in 2010 and 2011

| Source | DH | | | |  | RC-F_2_ | | | |
| --- | --- | --- | --- | --- | --- | --- | --- | --- | --- |
|  | DF | Sum of Squares | Mean Square | *P* value |  | DF | Sum of Squares | Mean Square | *P* value |
| SL |  |  |  |  |  |  |  |  |  |
| Genotype (G) | 260 | 644.03 | 2.48 | 0.00 |  | 231 | 256.71 | 1.11 | 0.00 |
| Environment (E) | 2 | 114.6 | 57.3 | 0.00 |  | 1 | 80.89 | 80.89 | 0.00 |
| G×E | 494 | 232 | 0.47 | 0.00 |  | 179 | 43.73 | 0.24 | 0.99 |
| SW |  |  |  |  |  |  |  |  |  |
| Genotype (G) | 260 | 644.03 | 2.48 | 0.00 |  | 231 | 73.65 | 0.32 | 0.00 |
| Environment (E) | 2 | 114.6 | 57.3 | 0.00 |  | 1 | 24.95 | 24.95 | 0.00 |
| G×E | 494 | 232 | 0.47 | 0.00 |  | 179 | 23.99 | 0.13 | 0.00 |

Table S2 The information of SSR markers linked with the QTL on A09 and C08 of *B. napus*

| Markers | Linkage  group | Primer sequences (5’→3’) | Position on R09 (Mb) | Position on O08 (Mb) |
| --- | --- | --- | --- | --- |
| CNU402 | A09 | F: GCCGACTCCTAGTGAGGAAA  R: TGTGTTTTGGGCTCAAAGGT | 26.67 | 27.23 |
| CNU296 | A09 | F: TCTCGTCGCTCTGAATTGTG  R: TTGTGAAATCAAAGCAAAAAGG | 30.76 | 31.71 |
| CB10373 | A09 | F: CGGTCAGATTCCAACAGA  R: TGTGTTTTGGGCTCAAAGGT | 30.80 | 31.75 |
| CNU114 | A09 | F: AGTCGGAGGAAACGCGAAATTA  R:CGAAATAAAGACAGACAGAGACATCCA | 31.28 | NA |
| CNU601 | A09 | F: TATGCCTCACCGTAAGATCG  R: GAGCTTTGTCTGGCATTGGT | 31.81 | 33.12 |
| YD097 | A09 | F: TTCGACAACGATGAGTTCCA  R: AGTGTTCGTGTTGCATGAGC | 31.81 | 33.12 |
| BRMS247 | A09 | F: TCTCTTTTGAGGTTTTCACTTTCAG  R: GCTGATTAAACTCTTACCACCAGAG | 32.99 | 34.65 |
| SWUC9 | A09 | F: CCTCGCTCTACCGTCAACAT  R: GAACAGCGAGGGACTCGTAG | 33.20 | 34.91 |
| CNU263 | A09 | F: GAGGAAGTACGGCAAGAAACCA  R: AGGACACATGTCCACATGAAAA | 33.7 | 35.58 |
| CEN070 | A09 | F: TACGTGGCGAAATGCTACTG  R: AGGATTACGTTTACACGGCG | 38.3 | 40.8 |
| ENA21 | C08 | F: GAGTGTTTGGAGCAGATGA  R: GGAGACTTTGCCTTTGTGT | 33.81 | 35.72 |
| SWUC11 | C08 | F: TCTGAAAATTCCGCTGAAGG  R: TGCTGGCTCATCTTCAGACA | 33.46 | 35.25 |
| SWUC10 | C08 | F: TATCAGCTGCGCTTCCTTTT  R: GGACGTGACGATGATGACAG | 33.38 | 35.15 |
| CNU356 | C08 | F: CGCATTTTCGCCGTCATTA  R: ACATCAGGCCGTCCCACTAA | 33.41 | 35.18 |
| CB10373 | C08 | F: CGGTCAGATTCCAACAGA  R: GCCATCTCAGAGACGACA | 30.80 | 31.75 |
| CY-04^a^ | A09 | F: AATGTCTTTGTAGGCTTCTGATATG  R: GGACTGTCAATCTTTGTGTCGCAT | 31.69 | 32.90 |
| CY-10^a^ | A09 | F: AGCCATCCCCCACTATATTACTCTG  R: TGGAATATCGGTATACACTAGCAAC | 31.78 | 33.07 |

^a^ region-specific SSR marker

Table S3 Information of the SNP markers with Δ (SNP-index) values exceeded the threshold in the peak region on chromosome R09

| SNP marker | Position on R09 (bp) | Δ (SNP-index) | Genotype in the ‘Large group’ | Genotype in the ‘Small group’ |
| --- | --- | --- | --- | --- |
| Bn-A09-p30836904 | 30836904 | 1 | T | C |
| Bn-A09-p30845744 | 30845744 | 1 | A | T |
| Bn-A09-p30860989 | 30860989 | 1 | G | A |
| Bn-A09-p30862870 | 30862870 | 1 | A | G |
| Bn-A09-p30893883 | 30893883 | 1 | A | G |
| Bn-A09-p30903184 | 30903184 | 1 | T | G |
| Bn-A09-p30914172 | 30914172 | 1 | A | G |
| Bn-A09-p30930982 | 30930982 | 1 | A | C |
| Bn-A09-p30958731 | 30958731 | 1 | A | G |
| Bn-A09-p30968893 | 30968893 | 1 | A | G |
| Bn-A09-p31007890 | 31007890 | 1 | G | A |
| Bn-A09-p31009450 | 31009450 | 0.6 | G | A |
| Bn-A09-p31010845 | 31010845 | 1 | T | C |
| Bn-A09-p31010864 | 31010864 | 1 | C | T |
| Bn-A09-p31021690 | 31021690 | 1 | G | A |
| Bn-A09-p31022310 | 31022310 | 1 | G | A |
| Bn-A09-p31213527 | 31213527 | 1 | A | G |
| Bn-A09-p31277719 | 31277719 | 0.67 | T | A |
| Bn-A09-p31299876 | 31299876 | 1 | C | T |
| Bn-A09-p31311597 | 31311597 | 1 | A | G |
| Bn-A09-p31467927 | 31467927 | 1 | G | T |
| Bn-A09-p31505718 | 31505718 | 1 | T | A |
| Bn-A09-p31517021 | 31517021 | 1 | C | A |
| Bn-A09-p31546401 | 31546401 | 1 | G | T |
| Bn-A09-p31572384 | 31572384 | 1 | C | T |
| Bn-A09-p31587679 | 31587679 | 1 | T | C |
| Bn-A09-p31622519 | 31622519 | 1 | C | T |
| Bn-A09-p31626590 | 31626590 | 1 | G | A |
| Bn-A09-p31631743 | 31631743 | 1 | C | T |
| Bn-A09-p31656047 | 31656047 | 1 | C | A |
| Bn-A09-p31706063 | 31706063 | 1 | C | T |
| Bn-A09-p31771680 | 31771680 | 1 | C | T |
| Bn-A09-p31778812 | 31778812 | 1 | C | A |
| Bn-A09-p31779289 | 31779289 | 1 | T | G |
| Bn-A09-p31784014 | 31784014 | 1 | T | C |
| Bn-A09-p31792898 | 31792898 | 1 | T | C |
| Bn-A09-p31806553 | 31806553 | 1 | C | T |
| Bn-A09-p31854011 | 31854011 | 1 | A | G |
| Bn-A09-p31870809 | 31870809 | 0.6 | A | G |
| Bn-A09-p31886042 | 31886042 | 1 | G | A |
| Bn-A09-p31918502 | 31918502 | 1 | G | T |
| Bn-A09-p31977508 | 31977508 | 1 | C | A |
| Bn-A09-p31978988 | 31978988 | 1 | C | A |
| Bn-A09-p31980306 | 31980306 | 1 | C | T |
